# Selections

**Published:** 1817-11

**Authors:** 


### PART III.

### SELECTIONS.

—— Factorum est copia nobis.

### FOREIGN WORKS.

### ANATOMY and PHYSIOLOGY.—*Monsters.*

Not long ago, an elaborate discussion, on the subject of monstrous productions in the animal kingdom, was published by Mr. Lawrence.* Herein, he has detailed some particular examples of monstrosity, enumerated the more frequent varieties of it in the human race, and attempted to trace to its source this mysterious aberration from the ordinary route of nature. He has been at great pains to convince us, that the development of the organs of the fœtus is influenced neither by the imagination nor mental commotions of the mother, nor by the impressions of external force, agents, to the operation of which they have been so often erroneously referred; that the lord of creation, humiliating as the reflection may be, is but a mere secretion from the vessels of the ovary; and that deviations from his usual form and structure are attributable to an irregularity of the powers concerned in generation. These positions few enlightened men will probably be found to controvert. But whether, by fully admitting them, any real progress be made in dissipating the deep obscurity which involves the generative process and its frequent aberrations, is a question naturally enough presenting itself. We think not. Terms do not invariably

* See *Medico-Chirurgical Transactions,* Vol. v. page 165.

imply knowledge; nor must they be received as a substitute for it. There is a bound in all the sciences at which the stride of human intellect is irresistibly arrested; and in the impenetrable region beyond that limit the mysteries of generation are for ever placed. The day is gone by, when, on the appearance of a headless or misshapen fœtus, the resentment of the Deity, the agency of imps and evil genii, or the machinations of the wizzard, were with equal confidence advanced and received as a satisfactory explanation of the phænomenon. And the philosopher now views these strange productions with interest only as they serve to elucidate some obscure problem in the science of physiology, and enable him to distinguish those organs, and the peculiar distributions of them, which are essential to the maintenance of extra-uterine life. Mr. Abernethy once examined the body of a child, in whom all the viscera of the thorax and abdomen were transposed. The heart occupied the right side, and the liver the left, of their respective cavities. In this curious case, the vena portarum terminated in the inferior cava, without traversing, as is usual, the substance of the liver: yet the gall-bladder contained some healthy bile. Thus, on the notice of Mr. Abernethy, was obtruded by the light of malformation, the obvious and important inference, to which no reasoning or practicable experiment could have conducted him, that the biliary secretion was not, as before supposed, invariably the product of venous blood.*

In that class of monstrous productions to which the term Acephali is, for the most part, indiscriminately applied, two very distinct varieties are found to exist. A trunk, without the slightest vestige of cranium or face, constitutes the one: in the other, the face itself is more or less perfectly developed; but the cranial vault, with its contained organ, defective or altogether wanting. To the former of these varieties Professor Chaussier has proposed to restrict the term Acephalus;† and to designate the latter by that of Anencephalus.‡ Thus, the confusion which has hitherto prevailed in anatomical writings, on the subject of headless monsters, will in future be obviated. This arrangement is adopted by the French anatomist, Beclard, who has recently published the first part of an interesting memoir on Acephali.§ He commences by some reflections on the singular error into which historians and travellers have fallen in describing whole nations of Acephali—an error, at-

* We have, five or six times, heard Mr. Abernethy mention this interesting fact in his lectures. It is not, we believe, recorded in any of his writings. Ed.

† From Α privative, and Κεφαλη, the head.

‡ From Α privative, and Εγκεφαλον, the brain.

§ *Mémoire de M. Beclard, sur les Acéphales. Première Parte. Bulletin de la Faculté de Médecine de Paris,* 1815—No. IX, and X.

tributable, in his opinion, to the influence of certain peculiarities of. dress, as in the Samoyedes; or to a natural depression of the head, which prevails among the inhabitants of the banks of the Caora, and causes the eyes to appear situated on the shoulders, and the mouth upon the breast. He then proceeds to cite from the works in which they are respectively consigned, numerous well-authenticated cases of acephali, with the precaution of, as frequently as possible, referring to the original description, in order to avoid the error in quoting for examples of acephali other varieties of malformation, and of adducing, as distinct facts, the same case differently recorded by several compilers. From this blunder, the celebrated Haller has not always been exempt. The "particular histories," constitute the first part of the Memoir; and from them M. Beclard proposes to "deduce the general history of acephali, which will be terminated by an explanation of this singular defect." The cases obviously do not admit of analysis. We shall probably revert to the memoir as soon as it is completed.

Another species of monstrosity, although very much more uncommon than that which we have just been contemplating, is occasionally observed. The head of a fœtus, possessing its full development, but wholly destitute of trunk and extremities, has more than once been expelled from the human uterus. A fact of this kind is stated in a late number of Hufeland's Journal.* It occurred in the practice of Dr. Elfes, of Neuss on the Rhine. It will be unnecessary to detail minutely the particulars of this extraordinary case; as they have been recorded in our Journal, by Dr. Von Embden. The figure of the monster, once in the possession of Dr. Elfes,† is illustrated by a clear engraving: and we are led to expect from Professor Rudolphi, that "more correct description, to which the case, viewed in its anatomical and physiological relations, is so justly entitled." Two teeth, belonging to the upper jaw, are represented in the engraving. The whole head has a very aged appearance.

We shall close our cursory review of this curious subject, by adverting to a case of monstrosity,‡ which constitutes one of the latest contributions of the lamented Italian anatomist, Mascagni, to that science, wherein he acquired such merited celebrity. It relates to a male fœtus, double in the superior parts and united below. One

---

* *Vorläufige nachricht und Abbildung eines höchst merkwürdigen monstrum, was einen blossen Kopfohne Rumpf und Arme darstellt. Journal der Practischen Heilkunde.* April, 1816.

† The labour had been superintended by a midwife; and consequently no very correct information could be obtained respectiug the singular phænomenon which it presented. She told Dr. Elfes that "the head was firmly enclosed in a bladder on one of the placentæ; which she opened and separated." See *Medico-Chirur. Journal,* vol. ii. page 167.

‡ *Giornale della Societá Medic. Chirurg. di Parma.* Tomo xv.

pericardium contained two hearts. Each of these had distinct lungs, two aortæ, and two superior venæ cavæ; but only one inferior cava. This, after having traversed the diaphragm, divided into two branches: one of which took a serpentine direction to the right, the other to the left. There existed also two thoracic ducts. Each passed along a vertebral column between the aorta and vena azygos; and terminated, as usual, at the angle formed by the subclavian and internal jugular veins. There was only one liver, but two spleens, and two stomachs with their intestines, which, towards the colon, re-united into one canal. The fœtus had two heads and two vertebral columns completely formed. The nerves and vessels displayed a size proportioned to the parts which they were destined to supply.

---

### II. PATHOLOGY and PRACTICE (*Medical*).

1. *Convulsive and Spasmodic Diseases.*—By the able researches of Dr. Saunders,* of Edinburgh, considerable light has recently been thrown upon the pathology and treatment of a family of diseases, frequently as intractable in the one respect, as obscure in the other. While urging him on to a farther prosecution of his labours in this new field of discovery, and wishing him the success and honourable compensation to which his exertions entitle him, we feel confident that we express the sentiments of all the more enlightened part of our profession in this country. Nor are we, without satisfaction, enabled to corroborate, in some degree, his important doctrines. The evidence about to be adduced, is, indeed, indirect as to its source; but, doubtless, it will possess additional weight, as furnished by a man to whom the views and opinions of Dr. Saunders seem to have been utterly unknown; and who evidently made and recorded the observation, with a mind uninfluenced by any peculiar theoretical notions. Had the subject of the fatal case of *tetanus*,† to which we allude, been inspected after death, by the scrutinizing eye of the Edinburgh pathologist, results much more decisive would probably have been obtained.

A workman, aged 28, robust, irascible, and much exposed to atmospheric vicissitudes, received, in the beginning of December, 1816, an injury on the left foot from a nail in his shoe. He took off the shoe and continued to work.—7th. Pain in the back: prevented by the wound from walking.—10th. Extraordinary sense of fatigue after exertion; slight stiffness of the jaws.—12th. Flexion of the neck and trunk difficult; appetite and sleep good; back painful. Continues his work; but on the morrow, after a good night,

---

* See the valuable papers by Dr. Moulson and Dr. Seeds, *Medico. Chir. Journ.* vol. iii. page 366, and 513; and Mr Webster's well detailed and most interesting Case of Rabies Canina, in our last Number.

† *Tetanos mortel produit par une cause tres-légère*, &c. Par M. Patissier, D. M. *Gazette de Santé*, Aout, 1817.

unable to quit his bed. Pain in the back increased : appetite unimpaired. Flexion of the head and body more difficult during the next night; the head bent back; the trismus aggravated; the muscles hard and contracted, but not painful on pressure; the motions of all the limbs perfectly free ; the head and abdomen without pain; tongue clean: violent constriction in the breast; skin hot; pulse full and frequent; deglutition of liquids much embarrassed. Venæsection, baths, and opium in frequent doses, were prescribed ; and from a crucial incision made in the foot escaped an ichorous and blackish pus. The pains increased towards evening, but the patient was relieved by a second bathing, of two hours' duration. Injections of laudanum, assafœtida and camphor, were administered. The night was restless; anxiety and tightness of respiration prevailed. In the morning, the trismus was stronger. The head and trunk appeared to form one piece. Respiration was stertorous; the skin covered with a clammy sweat. The face and tongue soon became of a violet hue; respiration slow, and the pulse small and frequent. Shortly after the application of leeches to the neck, and of a blister to the breast, the patient died.—On dissection, the internal membrane of the heart and large vessels was found of an intensely red colour; as was that portion of the arachnoid which lines the dura mater in the vertebral canal, and the membranous expansions by which the nerves are accompanied. The substance of the medulla was very much softened. The arachnoid of the cerebral ventricles, and that which covers the spinal marrow, presented but slight traces of inflammation.

By some of our countrymen, who have lately visited the medical schools of Paris, the fondness of the French for the red hot iron in the treatment of diseases, has been described in terms, which, if the subject were not such as to repress every feeling of this nature, might well excite a ludicrous idea. So numerous are the brandishers of these terrible instruments, that one cannot, without danger, pass through the wards of their hospitals, during the visit of the surgeons : and few diseases are denounced as completely hopeless, until they have been submitted to this fiery discipline. That the actual cautery exerts a very powerful, and occasionally, most beneficial influence on the animal organs, in a state of disease, there can be no doubt. But it is a cruel remedy ; and ought never to be employed, except in urgent and otherwise intractable cases, and where its success can be calculated upon with all the certainty of which our uncertain science will admit. The French, we suspect, are unjustifiably lavish, and indiscriminate in their application of it ; while the surgeons of this country have run into the opposite and equally reprehensible extreme of well nigh banishing from practice one of the most useful and unerring remedies of which the art can boast. The very impression which the hot iron, when applied to the human subject, must necessarily make on the patient's mind, is calculated to act beneficially; and will, perhaps, be adequate to effect a cure, where the malady consists in some irregular action, kept up by habit or morbid association, and wholly independent on organic lesion. Whether the immediate removal of the complaint, in the following case,* be attributable to the moral or physical operation of the remedy, may admit of doubt. From the suddenness of the result, we are disposed to refer it to the former.—A young girl had been tormented, for six months, with almost incessant *hiccup*. It ceased during deglutition, but re-appeared immediately after. The sleep was frequently disturbed. Antispasmodics and the warm bath had been employed in vain. In 1812, Dupuytren was consulted. He applied the actual cautery on the point corresponding to the tendinous centre of the diaphragm, and the hiccup immediately ceased. We should like to have tried, in this case, the effect of cupping and blistering in the vicinity of the origin of the cervical nerves; from the third, fourth, and fifth of which, it will be recollected, the phrenic nerve is derived.

To the treatment of *neuralgia* also, we consider it highly probable that the principles of Dr. Saunders might be advantageously applied. It is an affection which constantly exhibits, in its more aggravated forms, a decidedly spasmodic character; and is seldom, and certainly not in an essential manner, connected with any evident degree of pyrexia. We mean not here to enter into the controversy respecting the local or constitutional origin of neuralgia. Upon this point, however, the opinions of Dr. Armstrong are tolerably conclusive:† and it is singular enough, that his ideas relative to the cerebral source of the disease, and his experience of the efficacy of general depletion, and especially of "local blood-letting," and the application of blisters to the "scalp or to the nape of the neck," in its removal, go very far to countenance the conjecture which we have just started.

Our continental rivals seem to entertain no very correct notions on the pathology of neuralgia; and are consequently destitute of any fixed principles in its treatment. The invariable adoption of one particular plan or remedy in a disease, under all its various forms and complications, and loud assertions of the unerring success thereby obtained, if they beget no suspicion of the author's integrity, constitute a proof, stronger than which none can be adduced, that they, by whom such unqualified statements are implicitly received, possess no accurate or comprehensive views of the complaint in question. Meglin, a French writer, published, last year, a book,‡ the object of which is to recommend the employment of certain never-failing pills in cases of facial neuralgia; and we learn, from the various foreign Journals, that the practice, therein inculcated, is gaining ground in France. "The sedative remedy," says Meglin, "which I employ for the cure of tic-douloureux of the

---

* *Hoquets chroniques guéris, par l'application du feu. Gazette de Santé.* Aout, 1817.

† See his admirable "*Illustrations of Typhus*," page 212.

‡ *Recherches et Observations sur la Névralgie Faciale ou le Tic-Douloureux de la Face.* Par M. Meglin, D. M. &c. 8vo. Strasbourg, 1816.

face, is composed of extract of henbane and sublimed oxide of zinc. These two substances, mixed in equal parts, and formed into pills, have sufficed to remove the complaint: yet I add to them the extract of wild valerian, in order to give the medicine a tonic virtue, without diminishing its sedative properties; especially in those cases where, from the inveteracy of the disease and the violence and frequency of the paroxysms, the exhausted patient stands in need of recruiting. On the same principle, I employ cinchona alternately with the pills." M. Meglin cautions those who wish to try his remedy, to use such extract of henbane only as is recent, and has been prepared by a sand-bath. Twelve cases are detailed in illustration of the success of this practice. We subjoin a concise sketch of one of the most strongly marked and shortest.

A man, aged 34, of bilio-sanguineous temperament, was attacked, some years ago, with periodical pain in the face. It commenced, with the rapidity of lightning, in the centre of the right eye-brow, and descending obliquely, fixed on the ala and middle of the nose. It came on every morning at seven o'clock, increased in violence till towards ten, and subsided about one P. M. In February, 1816, the pains, which had a lancinating character, were become so dreadful, that the patient could scarcely answer the questions put to him; and he declared, in his more tranquil moments, that, unless his sufferings were relieved, he would destroy himself. A violent fit of anger, into which he had fallen just before the invasion of the disease, was the only evident cause to which it could be referred. The paroxysms were so distinctly marked, and during their absence the patient felt himself so well; and the parts affected by the pain were so perfectly destitute of any morbid sensibility, that it was resolved to treat the case as an intermittent fever. The cinchona, taken for three days, had only the effect of rendering the pain continual. One of M. Meglin's pills was then directed to be taken night and morning, with a glass of infusion of orange and lime flowers; and the dose of the former to be gradually increased to five. Upon taking this number, the pain sensibly diminished, and soon wholly disappeared. The patient persevered for eighteen days in the use of this remedy, and has since suffered no return of his complaint.—The pills employed by M. Meglin contain, each, three grains of the mixture specified. We have yet had no opportunity of trying their effect. Some farther notice of this work will probably be taken in our analytical department.

The partizans of the opinion, that neuralgia is primarily and essentially a local disease, adduce, in support of this doctrine, examples, in which its removal has been accomplished by division of the morbid nerve, or some other mean of purely topical operation. But we believe, that, on minutely tracing the history of such cases from their origin to their close, the cure, effected by local remedies, will either be discovered not to have been permanent; or the success, ascribed to such remedies, proved to have really resulted from previous constitutional treatment. A case of " cure of suborbitar Neuralgia, by the external use of acetic æther,"* communicated by a foreign writer, very aptly illustrates our argument. We shall briefly transcribe it, as, moreover, confirming our notions on the cerebral origin of the malady. In the beginning of last April, a man, aged 27, sober and robust, of short stature and sanguine temperament, constantly exposed to a current of air in a low situation, complained of vertigo and drowsiness. The countenance was heavy, the conjunctiva injected, and the pulse hard and full. Bleeding afforded relief; but the symptoms shortly reappeared. Stimulant pediluvia, and light aromatic infusions, produced no effect. A blister was applied to the nucha, and leeches to the temples. The head was relieved; but an attack of suborbitar neuralgia, of the right side, soon ensued, which, after having resisted all the usual remedies, was completely cured, on the ninth day, by one external application of acetic æther.

Tic-douloureux of the foot, appropriately termed Plantar Neuralgia by Professor Chaussier, constitutes a very rare variety of the disease. That experienced practitioner has met with but one example of it. Dr. Marino,† a Piedmontese physician, suffered an attack of this painful affection in early life; and, although he attained an advanced age, never succeeded in perfectly removing it. Latterly it was complicated with convulsive asthma. Previously to the invasion of the neuralgia, the Doctor had repeatedly suffered from lumbago and sciatica. On the relief of the latter by topical stimulants, the foot of the opposite side became insensible, cold, motionless, and began to waste. After the long continued employment of vapour-baths, sensation and an imperfect degree of motion were restored, and the tarsus displayed the appearance of erysipelatous inflammation. In the following spring, from the use of the mineral waters of Vivadio, the foot nearly regained its healthy condition: but the nails separated from four of the toes, and a slight degree of numbness returned. A year after this, the waters of Vivadio were again had recourse to with speedy success; for the sciatic pain, which had shifted from the thigh to the leg, and now the external part of the foot, first became affected with neuralgia. The pain had a spasmodic, lancinating, vibratory character, and gradually grew more constant and severe. Of all the remedies employed against it, constriction with a tight bandage was most

---

* *Guérison d'une Névralgie sous-orbitaire, par l'usage extérieur de l'éther acétique.* Gazette de Santé, Juin 1817.—Acetic æther is prepared by distilling three times over a mixture of equal parts of alcohol and acetic acid, and condensing the vapour by means of a receiver placed in a refrigerant bath. The product of the third distillation is a mixture of acetic acid and æther. After having saturated the acid by potash, distillation is again to be performed by a gentle heat; and that, which then passes over, is acetic æther. *Dictionnaire des Sciences Médicales,* Article *Æther.*

† *Maladie spasmodique de la plante des pieds.* Gazette de Santé, Aout 1817.

efficacious. In the third year from its commencement, the patient sustained a paroxysm so sudden and severe, that he fell down, and, on being carried to bed, was seized with general convulsions, which lasted eighteen hours. From this time, the foot was indurated, hot, and dry, with corrugation of the skin. A sense of tingling sometimes preceded the paroxysm: sometimes it commenced abruptly and without prelude. It subsided with the rapidity of an electric shock, and recurred with an universal spasm of the muscles. The pulse was then accelerated, small, and feeble; the urine light, frequently voided, and scanty; the bowels obstinately costive. Occasionally subsultus of the tendons of the foot, and palpitation of the popliteal muscles, were observed. The paroxysm lasted from twelve to thirty hours; and the shocks of pain often insensibly diminished in force and frequency. On its decline, the pulse became more soft and slow, and the urine more high coloured; the foot resumed a genial warmth, and was covered with moisture. Long and tranquil sleep succeeded; and on the morrow a sort of numbness in the integuments of the foot only was felt, without any relic of pain, spasm, or debility. An unpleasant sensation was induced by slight pressure or friction. Forcible compression on the sole of the foot excited considerable heat. The pain sometimes remained dormant during a year or more. Sometimes it returned at intervals of from fifteen to thirty days. In the more violent and protracted paroxysms, the patient was additionally tormented with severe priapism, or an ardent urethral discharge. Dr. Marino has himself communicated an account of this case, which, to the unprejudiced mind, will, we think, afford tolerably clear evidence of the constitutional origin and nature of neuralgia.

2. *Paralysis.* The supervention of convulsions on paralysis has frequently been found productive of the happiest consequences. Availing himself of this observation, M. Fouquier, a French physician, resolved on trying the effect of convulsious, artificially induced, in paralytic cases. With this view, he selected the nux vomica, the poisonous action of which has been proved to consist in the production of tetanus. An account of his first experiments with this formidable remedy was communicated to the *Société de la Faculté de Médecine*, in 1811; and, in the recen ly published memoir* now before us, he details the result of his subsequent experience in its administration: and this, he assures us, has been very generally successful. Many of the paralytics, subjected to his treatment, have been completely cured. Some again have experienced only partial relief; but " none could accuse the nux vomica of having aggravated his disease." Dr. Fouquier hastens, therefore, " to announce to physicians a new specific; to present some

---

* *Mémoire sur l'Usage de la Noix Vomique dans le Traitement de la Paralysie.* Par M. Fouquier, &c.—*Bulletins de la Faculté de Medecine, &c* 1816, 1817;—and *Journal Universel des Sciences Médicales,* Janvier, 1817.

remarks on the singular effects of the medicine, and to point out some rules in its exhibition." And he calls on all those, who are interested in the progress of the science, to unite with him in "fixing the character of a substance, as curious in its mode of operation as important from its virtues." Sixteen cases, illustrative of the employment of nux vomica in paralysis, are then minutely detailed. Of this number, eight are said to have been treated with perfect success; and, in the majority of the remainder, considerable relief appears to have been obtained. The fourth history, as one of the most interesting, we shall transcribe for the information of our readers.

A porter, aged 50, of robust constitution and generally healthy, except that, some years before, he had thrice suffered from pulmonary or pleuritic inflammation, was, in February, 1814, exposed to very cold rain on a fatiguing journey. What influence this accident might exert on the disease which speedily followed, is uncertain; but, from that time, the man complained frequently of general lassitude, which he attributed to excessive labour. He often felt head-ach; and was sometimes seized with slight dazzling. In May, his hands began to fail, and were no longer completely under the influence of volition. The inferior extremities also soon became weak in a still more remarkable manner. Both sides were equally affected, and the man was confined to his bed, without the power of moving. The limbs grew daily more emaciated, but the weakness continued the same. In June, he was admitted into *la Charité*. He had been bled in the commencement of his disease, and attributed his increasing weakness to this operation. In the hospital, he was blistered on the lumbar region, rubbed with stimulating liniments, and took tincture of cantharides in a sudorific potion. This plan was continued for a fortnight without effect. The extract of nux vomica was then administered in pills, of two grains each. One pill was given at a dose for the first ten days; then two till the commencement of August, when Fouquier himself undertook the treatment. The medicine hitherto had merely excited a sense of prickling in the limbs; yet the will seemed to have acquired more command over the muscles of the diseased parts. Still, however, the man could neither move his legs, nor use his hands to feed himself. The medicine was now pushed gradually to the amount of twelve grains a day. A tetanic rigidity of the limbs and trunk then came on, with heat and disorder of the head. This affection was painful, and recurred with each fresh dose of the nux vomica. It gradually extended to the paralysed muscles, accompanied with prickling of the legs, and went off by degrees in about an hour. On increasing the dose of the remedy, transient startings were induced in addition to the wonted rigidity. From these shocks on the muscular system, there was a daily increase in the power and facility of motion; but the limbs were still emaciated. In September, the patient was able to cut his bread and feed himself. Towards the close of the month, a general spasmodic rigidity, most strongly marked in the lower limbs, was determined by a more frequent exhibition of the remedy. This tetanic affection, accompanied with dyspnœa, sweats, and anxiety, continued three hours; and was succeeded by a new accession of strength and agility. In October, the man could walk unsupported, but with a tottering step. There was subsequently little alteration: for, although his stick was laid aside, the patient's gait continued vacillating and his limbs stiff. The progress of the cure was entirely suspended by the winter's cold. Both feet swelled; and a corn, situated on the left, prevented walking. From the discontinuance of M. Fouquier's visits, and a failure in the supply of the nux vomica, convalescence was considerably retarded.—On the 28th of June, 1815, the patient left the hospital, with his limbs somewhat stiff, but capable of performing all their natural movements.

All the other cases brought forward by M. Fouquier, appear to have been detailed with great fairness and candour: and it is but an act of justice to remark, that many of them afford instances of recovery much more perfect than that which we have selected. It is, however, one of those which most strikingly illustrates the singular operation of the nux vomica. The remedy was always administered either in the form of powder or of the alcoholic extract. We cannot, from a careful perusal of the cases, descry any difference of operation or result in these two modes of exhibition. Four grains of the powder, or two of the alcoholic extract, may be given from three to six times a day. But it is better to begin with small doses, and repeat them at long intervals, so that the action of the remedy may be vigilantly observed. Sixty-four grains of the former preparation, and twenty-four of the latter, appear to have been the largest quantities of the substance administered in one day. The watery extract is deliquescent, but Fouquier thinks that, in a mixture of this and the alcoholic, all the active properties of the drug would be combined. In one case, it was employed with great success in the form of a clyster. Half an hour commonly elapses ere the medicine begins to operate; and, what is inexplicable by any of the known laws of physiology, it seems to determine a spasmodic contraction of the paralysed muscles, without implicating the sound parts; and to act more powerfully on those muscles in proportion as they are more completely destitute of sensibility and motion. Equally incomprehensible is it, why the spasm, induced by nux vomica, should be accompanied by extension of the abdominal and flection of the thoracic limbs. A constant effect of this remedy is that of increasing the appetite, and diminishing the frequency of alvine evacuation. Sometimes it induces a sort of intoxication; and, when rashly administered, general tetanus, with all its train of frightful and distressing symptoms. In one paralytic, an affection of the encephalon, resembling delirium, resulted from its employment; and several were compelled to suspend the use of the remedy from the sense of burning heat in the stomach, consequent upon its ingestion. We regret our inability to give, at present, a more detailed account of this important memoir. Many a painful disappointment has taught us to view novel and extraordinary remedies with a suspicious eye. British practitioners, however, will not, we hope, be tardy to repeat the experiments of M. Fouquier. And if the effects of the nux vomica upon a disease so frequent and afflicting, and commonly so hopeless as paralysis, prove really to be such as he has described, we shall hail and revere that gentleman as one of the greatest friends and benefactors of the human race. Even the illustrious Meglin himself, must resign his *Saltem non omnis moriar,** to encircle the honoured name of Fouquier.

3. *Intestinal Affections.* Notwithstanding the very clear and masterly manner in which the pathology of puerperal fever has been elucidated by our countrymen,† many of the best continental writers still continue to propagate incorrect or erroneous notions on this subject. Gastellier, of whose work‡ we intend, ere long, to present a brief notice, denies the existence of puerperal fever as a distinct and essential malady. And even Robert, who has added to his publication on cancer§ an appendix, wherein he professes to exhibit some " new physiological views on the nature and treatment" of this disease, and who seems to have acquired a tolerably correct knowledge of its seat and general character, is evidently far behind us in the road of practice. A cursory sketch of this memoir will not prove uninteresting to the reader; and the introduction of one of Dr. Robert's cases will fully demonstrate the accuracy of our criticism. Previously to entering upon a general description of the disease, and characterizing its several species, of which he distinguishes no fewer than seven,|| Dr. Robert cites several cases from the writings of Hippocrates, Willis, White, and Gastellier, and records eight from his own practice, in order to determine precisely its pathognomonic symptoms. He then reviews the opinions of different authors who have written professedly on it; exhibits its general description, with a notice of all the signs which precede or accompany its fortunate or fatal termination, and communicates the result of a chemical analysis of the fluid effused into the peritoneal cavity of those who have perished from it. Lastly, he states his own ideas on the true denomination and nosological arrangement of

---

* The inventor of the anti-neuralgic pills has the modesty somewhat broadly to insinuate, that he considers this motto applicable to himself.—See " *Rech rches et Observations.*" P 121.

† *See* the valuable works on puerperal fever, not long ago published by Dr. Armstrong and Mr. Hey junior, of Leeds.

‡ *Des Maladies aigues des Femmes en couche*, 8vo. Paris, 1811.

§ *L'Art de prévenir le cancer au Sein*, &c. 8vo. Paris. 1812.

|| 1. *Fièvre puerpérale simple ou péritonite.* 2. —— *puerpérale gastrique.* 3. —— *puerpérale intermittente, simple.* 4. —— *puerpérale adynamique.* 5. —— *puerpérale milliaire.* 6. *puerpérale ataxique.* 7. —— *puerpérale rhumatismale.*

this fever, and concludes with an exposition of the methods employed by different authors for its cure.

The treatment adopted in the following strongly-marked case, will be regarded as frivolous and inert by those who have witnessed the happy effects of Dr. Armstrong's depletory practice. A lady, aged 35, very thin, and of delicate constitution, had, during pregnancy, suffered from domestic troubles. Her parturition was laborious; and, on the first day, she was seized with slight shivering, fever, and intense head-ache. The lochia did not appear, and the abdomen was traversed by lancinating pains. On the 2d, the pain and fever were aggravated. The abdomen was swollen and painful; the pulse ninety in a minute. Emollient fomentations, and an injection of bran-water and decoction of madder, were prescribed; but neither general nor local blood-letting. On the 3d, the head was affected with slight delirium, and the patient screamed. The abdomen assumed an uncommon degree of sensibility, and the pulse rose to one hundred and fifteen. Fomentations and clysters continued. The urine was scanty, crude and limpid. The delirium increased. The whole system was convulsed by subsultus tendinum. Camphor and opium were administered. Violent hysterical symptoms ensued. The stools, evacuated by the clysters, were blackish, and resembled goat's dung.—4th. Abolition of the senses, carphologia; pulse extremely hurried, and sudden cessation of the abdominal pain. On the 5th the patient expired.—Dissection. Four ounces of serum were found effused at the basis of the brain. The thorax was sound. The abdomen filled with a purulent sanies; the peritonæum and omentum inflamed and specked with gangrenous points. Albuminous flocculi swam in the peritonæal serum. Not one drop of milk was found in the breasts.

Walter, who has inspected the bodies of more than five thousand women dying in child-bed, invariably found, in puerperal fever, the peritonæal covering of the intestines, of the uterus and its appendages, the omentum, mesentery, and peritonæum lining the abdominal and pelvic cavities, inflamed, and smeared with a pus-like matter. This exudation he compares to that which takes place from the pleura in consequence of inflammation. He never found, on these occasions, the mucous or muscular structure of the uterus implicated.

After the specimen which we have just given of Dr. Robert's practice, in puerperal fever, it will be unnecessary to detail particularly his opinions upon its treatment. Emetics, venæsection, abstraction of blood by leeches from the vulva, opium, glysters, and fomentations, constitute the remedies on which he principally relies. His analysis of the peritonæal fluid will be more correctly included under another division of our Selections.

---

* From καρφος, straw, and λεγω, to catch; a motion of the hands, often preceding death, particularly in typhoid fevers.

In the ensuing case of "fatal diarrhœa in a puerperal female,"* taken from a German writer, the traces of neglected uterine and peritonæal inflammation may be clearly descried. A young person in the seventh month of her first pregnancy, while in the act of carrying home a load of wood upon her head, was prematurely delivered; and, from want of proper help, lost much blood, and was considerably reduced. In consequence of bad diet and nursing, she was seized with dreadful pains in the abdomen and copious diarrhœa. On the eleventh day from the accident, she was admitted into the hospital; but, it was then too late: she sank in a few days. On dissection, the intestines were found inflamed throughout, and covered with a firm white membrane, particularly the colon and rectum. This membrane was, in some places, as soft as the pellicle of cream. The pouch between the bladder and uterus was filled with a ropy, glutinous, jelly-like, whitish fluid. The uterus was of a volume equal to that which it commonly exhibits between the second and third month of pregnancy. Its orifice was yet open and much inflamed, and in the cavity of the organ a portion of the placenta still firmly adhered.

We may here not inappropriately cite an instance of "strangulation of the small intestine, terminating in fatal gangrene;"† which, perhaps, from the curious mode of production of the stricture, is unprecedented in the annals of medicine. The subject of the case, aged 19, and previously healthy, was seized with slight colic, which gradually became violent. On the second day, the abdomen was somewhat sore; the pulse much accelerated, full, and regular; the countenance quick. There was acute fever. Vomiting of green matter ensued: the night was restless. On the third, the man had the *facies hippocratica*, irregular intermittent pulse, tumified abdomen, with great pain about the navel, and cold and livid extremities. He expired while vomiting a deep green and extremely offensive fluid; the discharge of which continued some time after death. The only remedies employed were fomentations and oily glysters.

On dissection, the abdomen was distended with offensive gas; and the peritonæum contained a large quantity of fœtid blackish serum. There were several gangrenous spots on it and the large omentum. The small intestines were inflamed throughout, and partly gangrenous. The gangrenous portion of ileum (of which the minute description given by MM. Beclard and Cloquet is transcribed below) was situated to the right of the hypogastrium before and behind the psoas muscle. The cæcum, with its appendix, the colon and rectum, were only slightly inflamed. The internal membrane of the stomach was not implicated.

---

* *Beobachtung eines tödlichen Durchfalls bei einer Kindbetterin.—Journ. der Pract. Heilkunde,* Marz, 1816.

† *Observation sur un étranglement de l'Intestin grêle, terminé par la gangrène et la mort.* Par M. Bougon, &c. *Bulletin de la Société de la Faculté de Médecine, &c.*

*Description of the strangulated Portion.* On loosening the very tight knot which constituted the strangulation, a moveable appendix, six inches long, was seen rising from the ileum. It had encircled the intestine and its mesentery, in such a manner as to form a real knot around them. The loop of ileum, thus strangulated, was a foot and a half in length. At the point from which the appendix originated, the calibre of the intestine was somewhat contracted. Of the two ends of the intestine situated above the knot, the superior was red, inflamed, and much dilated; the inferior pale, contracted, and without trace of inflammation. The strangulated portion was red, much dilated, and contained about two pints of black, fœtid, and very fluid blood. The appendix was full of a similar liquid, which could not be forced into the intestine on account of the obstacle opposed to its passage by the knot. The membranes of the gut were red, and, as it were, injected with blood. The knot formed by the appendix must have been recent, for it had made no (permanent) impression on the intestine, which resumed its (ordinary) calibre, as soon as this accidental ligature was removed.

Pathology and Practice *(Surgical).* Rupture of the patella, or of the strong tendon by which it is connected to the tubercle of the tibia, is no uncommon consequence of inordinate contraction of the great extensor crural muscles. But laceration of the *superior* ligament of the patella, or, more correctly speaking, of the tendon of the extensor muscles inserted into that bone, is an accident which we have never met with in practice; and which, we believe, has not till lately been noticed in surgical writings. Within the last year, however, two examples of it have been observed, and communicated by reputable French authors. The first, given on the authority of Professor Dupuytren,* we shall briefly trace.—A middle-aged man, while running with his utmost speed, threw himself, under the apprehension of falling, violently backward. Instantly he felt a severe pain in the knees, and fell, without the power of rising. He kept his bed for some months; and afterwards, on attempting to walk, found upon the front of the left thigh, about two thirds down it, a soft, painless, and transverse tumour; which disappeared when the limb was extended and at rest, and returned during its contraction. While resting on the right leg, in an attempt to mount his horse, he felt a severe pain on this side, and was obliged to lie in bed two months longer. A swelling, like that of the other, was now discovered on the right thigh. Dupuytren was consulted; and observing, with the phenomena which have just been noted, a depression at the point where the superior ligament of the patella commonly projects, declared at once the nature of the injury. The second

---

* *Fracture des ligaments supérieurs des deux rotules. Gazette de Santé,* Novembre, 1816.

instance* occurred to an elderly man, in an effort to save himself from a fall, while descending stairs with rapidity, In this case, also, both tendons were ruptured. The accident was at first mistaken, and consequently maltreated ; but, two months afterwards, M. Barbieux, with some difficulty, recognized it by the narrative of the patient, and the "transverse groove existing above the two patellæ." The appearance of this groove, superadded to the common symptoms of transverse fracture of the patella, will, we apprehend, constitute a sufficient ground of diagnosis in such cases. When the nature of the accident has been at first mistaken, and the time for attempting reunion of the ruptured tendon is irrevocably gone by, as in the examples just recorded, all that the surgeon can accomplish is the support of the limb by artificial means, so as to furnish the power of standing and progression, otherwise nearly impracticable. This object Barbieux proposes to attain by an apparatus which consists of two strong calf skin knee-caps, buckling externally on the lower extremity, and embracing the superior third part of the leg and inferior third part of the thigh, and of a strap, equal in breadth to the rectus femoris muscle, attached below to that part of the knee-cap corresponding to the inferior extremity of the patella, and above to a waistband supported by strong braces. The modern treatment of fractured patella, by permanent extension of the leg upon the thigh, and flexure of the thigh upon the pelvis, would be obviously applied to *recent* accidents of this nature. Yet, in the opinion of the French writer, maintenance of the separated parts in contact would be more difficult in the latter than in the former case.

III. CHEMISTRY *(Animal).* Dr. Robert,† of Marseilles, has analysed the fluid effused into the peritoneal cavity of women dying from puerperal fever ; and has found it, in every instance, to possess a perfect chemical identity with that furnished by the inflamed pleura. The peritoneal fluid, after depositing a copious whitish precipitate, which afforded albumen to the different reagents, was of a clear yellowish white colour, and had the property of turning green the syrup of violets. But the nature and proportion of the alkali enduing it with this property, appears not to have been ascertained. The flocculi, which swim in the abdominal serum of puerperal subjects, have been regarded as a cheesy substance, formed of the coagulum of the milk. This opinion is refuted by Dr. Robert. Ammonia, mixed with the substance in question, scarcely acted upon it as a solvent ; and evaporation developed in it all the characters of albumen.

---

* *Rupture des deux muscles ilio rotuliens.* Par M. Barbieux, &c. *Bulletin de la Société de Médecine Pratique de Montpellier.—Histoire et Mémoires de la Société,* &c. Janvier, 1817.

† *Appendice—L'Art de prévenir le Cancer,* p. 419.

CHEMISTRY *(Vegetable)*. A faint outline of the result of some experiments upon ipecacuanha, by Magendie and Pelletier, has already been communicated in this Journal.* We deem it a duty to lay before our readers a more detailed account of their interesting "chemical and physiological researches" † upon this root. These researches were confined to its three most common species: the brown ipecacuan *(psychotria emetica)*; the grey *(calicocea ipecacuanha)*; and the white *(viola emetica)*.

The powder of all three, submitted to the action of cold and warm sulphuric æther, rectified alcohol, and distilled water, in succession, furnishes several principles in various proportions. The most remarkable of these is a substance, which, when dried, appears under the form of reddish brown transparent scales, possessing somewhat of the odour of brown sugar, and a bitter, acrid, but not nauseous taste. Boiling water has no action upon it. The most simple method of obtaining it is to treat powdered ipecacuan with æther at 60°, so as to separate an oily odorous matter which it contains. When the ipecacuan yields nothing more to the æther, alcohol should be poured upon it; the tincture, thus formed, be evaporated by a sand-bath; and the residuum be re-dissolved in cold water. Thus it abandons the wax and little oily matter still mixed with it Maceration on carbonate of barytes separates its gallic acid. Again treated with alcohol, and evaporated to dryness, the substance is in a state of purity.

Pelletier thinks that this substance, which he has found in the vomitive plants of different families, should rank among the immediate principles of vegetables, under the name of *émetine*; which alike indicates its most remarkable property, and the plant in which it was first discovered.

'Emétine possesses, in a very high degree, the vomitive property; and, from the circumstance of its having neither nauseous taste nor smell, and of its perfect solubility in water, is preferable to ipecacuan in substance. In the quantity of from two to four grains, given at two or three times, it may be employed as an emetic; but it cannot be prescribed, like ipecacuan, in stronger doses, with impunity. Ten grains have induced, in dogs, protracted vomiting, diarrhœa, and fatal drowsiness ‡ Inflammation of the lungs and intestinal canal was found after death. The action of this substance may be at once arrested by exhibiting an aqueous solution of nut-gall, which immediately combines with it. The "conclusions" of these gentlemen we need not repeat. They obviously result from the facts just developed.

---

* *Medico-Chirurg. Journal*, vol. iii. p. 530.

† *Recherches Chimiques et Physiologiques, &c.*; par MM. Magendie et Pelletier. *Annales de Chimie, &c.* 1817. *Gazette de Santé*, Juillet, 1817.

‡ It possesses a decidedly narcotic property.

One hundred parts of the *cortical* powder of the *three* species of ipecacuan, and of the *ligneous* portion of the *brown*, were found to afford the following products:

| | Brown Ipecac. Cortical Portion | Grey Ipecac. Cortical ----- | White Ipecac. Cortical ----- | Brown Ipecac. Ligneous ----- |
|---|---|---|---|---|
| Fat oily matter . . . . . . . . | 2 | 2 | — — | slight traces |
| Vomitive — (Emétine) . . | 16 | 14 | 5 | 1, 15 |
| Vegetable wax . . . . . . . | 6 | — — | — — | — — |
| Gum . . . . . . . . . . . . | 10 | 16 | 35 | 5 |
| Starch . . . . . . . . . . . . | 42 | 18 | — — | 20 |
| Woody matter . . . . . . . . | 20 | 48 | 57 | 66, 60 |
| Vegeto-animal — . . . . . | — — | — — | 1 | — — |
| Extractive — (not vomitive) | — — | — — | — — | 2, 45 |
| Gallic acid . . . . . . . . | slight traces | — — | — — | slight traces |
| Loss . . . . . . . . . . . . | 4 | 2 | 3 | 4, 80 |
| | 100 | 100 | 100 | 100 |

Hence it appears, that *brown* ipecacuan contains the greatest proportion of emetine, and is the most active; and that the *ligneous* portion, in consequence of the small quantity which it affords, ought to be rejected from all preparations of this medicine.

We shall take an early opportunity of preparing, and putting to the test, this newly discovered and valuable substance. EDIT.

